# Rhizophagy Cycle: An Oxidative Process in Plants for Nutrient Extraction from Symbiotic Microbes

**DOI:** 10.3390/microorganisms6030095

**Published:** 2018-09-17

**Authors:** James F. White, Kathryn L. Kingsley, Satish K. Verma, Kurt P. Kowalski

**Affiliations:** 1 Department of Plant Biology, Rutgers University, New Brunswick, NJ 08901, USA; kathryn.l.kingsley@gmail.com; 2 Centre of Advanced Study in Botany, Banaras Hindu University, Varanasi, UP 221005, India; skvermabhu@gmail.com; 3 U.S. Geological Survey, Great Lakes Science Center, 1451 Green Road, Ann Arbor, MI 48105-2807, USA; kkowalski@usgs.gov

**Keywords:** endobiome, endophyte, nutrient transport, reactive oxygen, rhizosphere, symbiosis

## Abstract

In this paper, we describe a mechanism for the transfer of nutrients from symbiotic microbes (bacteria and fungi) to host plant roots that we term the ‘rhizophagy cycle.’ In the rhizophagy cycle, microbes alternate between a root intracellular endophytic phase and a free-living soil phase. Microbes acquire soil nutrients in the free-living soil phase; nutrients are extracted through exposure to host-produced reactive oxygen in the intracellular endophytic phase. We conducted experiments on several seed-vectored microbes in several host species. We found that initially the symbiotic microbes grow on the rhizoplane in the exudate zone adjacent the root meristem. Microbes enter root tip meristem cells—locating within the periplasmic spaces between cell wall and plasma membrane. In the periplasmic spaces of root cells, microbes convert to wall-less protoplast forms. As root cells mature, microbes continue to be subjected to reactive oxygen (superoxide) produced by NADPH oxidases (NOX) on the root cell plasma membranes. Reactive oxygen degrades some of the intracellular microbes, also likely inducing electrolyte leakage from microbes—effectively extracting nutrients from microbes. Surviving bacteria in root epidermal cells trigger root hair elongation and as hairs elongate bacteria exit at the hair tips, reforming cell walls and cell shapes as microbes emerge into the rhizosphere where they may obtain additional nutrients. Precisely what nutrients are transferred through rhizophagy or how important this process is for nutrient acquisition is still unknown.

## 1. Introduction

It is widely known and accepted that most plants obtain nutrients generally through absorption of dissolved inorganic nutrients from soils [1]. However, it is also known that some plants engage in nitrogen-transfer symbioses where plants associate with prokaryotes that fix nitrogen in association with roots and transfer that nitrogen to plants [2,3]. Among these nitrogen-transfer symbioses are actinorhizal symbioses that occur in three orders of plants (*Fagales*, *Rosales* and *Cucurbitales*) where roots may become inter-cellularly and intra-cellularly colonized by diazotrophic actinomycetes of the genus *Frankia* that inhabit nodules in roots [2]. Families of plants where actinorhizal symbioses are common include: *Betulaceae*, *Elaeagnaceae*, *Fagaceae*, *Myricaceae*, *Rosaceae* and so forth [4]. Other nitrogen-transfer symbioses are the rhizobial symbioses where certain diazotrophic bacteria infect root hairs and move into the root cortex where they become intracellular and stimulate formation of nodules; bacteria then situate in the cytoplasm of nodule cells in vesicles and transfer nitrogen to plants in the form of ammonia [5,6]. Rhizobial symbioses are limited principally to legumes (family *Fabaceae*) [5]. In some plants, diazotrophic cyanobacteria form nitrogen transfer associations with plant tissues where they fix nitrogen and transfer it to the plant [4]. Among these are species of the genus *Gunnera* that possess specialized glands that secrete polysaccharides to attract cyanobacteria, which enter into tissues of the stem and become intracellular within host cell vesicles where they fix nitrogen that is subsequently transferred to the host plant [4].

In all the previously discussed nitrogen-transfer symbioses, hosts evolved ways to internalize and engage in prolonged symbiosis with diazotrophic prokaryotes using specialized plant symbiosis organs or tissues. All of these associations are restricted to specific families that evolved specialized symbiosis organs (nodules or glands); however, all species of plants internalize microbial endophytes into plant tissues that do not involve specialized organs [7,8,9,10,11,12,13,14,15,16,17,18]. Microbial endophytes have been shown to provide a plurality of benefits to host plants, including growth promotion, improved biotic and abiotic stress tolerance and increased disease protection [19,20,21,22,23,24,25,26,27,28,29]. One logical means of plant growth promotion by plant endophytes is improved nutrient acquisition by plants. However, mechanisms for direct nutrient transfer from endophytic bacteria to plants have been elusive [30,31]. In some cases, bacteria that have not been found to be capable of fixing atmospheric nitrogen are nevertheless found to scavenge nitrogen and other nutrients from the rhizosphere and transfer them to plants [32]. In other cases, endophytes have been shown to increase solubilization of bound phosphates in the rhizosphere, and thus have been hypothesized to function by increasing plant phosphate supply in the rhizosphere [17]. In absence of a mechanism for direct transfer of nutrients from microbes to plants, many scientists attribute growth promotion largely or partially to effects of microbe-produced phytohormones, disease control, or other non-nutritive benefits [33,34,35,36,37,38].

Evidence for a mechanism for direct transference of nutrients from symbiotic microbes to plant roots was provided by Paungfoo-Lonhienne et al. [39]. Through a series of experiments, these investigators showed that plant roots (*Lycopersicum esculentum* and *Arabidopsis thaliana*) internalized bacteria and yeasts into root cells where microbes appeared to be degraded in time. Paungfoo-Lonhienne et al. [40] later denominated this microbe internalization and degradation process ‘rhizophagy’ to denote that roots appeared to be ‘eating’ microbes. Adamczyk et al. [41] and Paungfoo-Lonhienne et al. [42] demonstrated that plants had the capacity to employ secreted proteases in order to degrade proteins, further supporting the hypothesis that plants actively degrade microbes and their protein products associated with roots. White et al. [43] documented degradation of bacteria on root surfaces as a result of the action of root-secreted reactive oxygen and proposed that through the action of reactive oxygen roots may be scavenging nitrogen from bacteria that colonize roots. White et al. [44] later documented internalization of bacteria into periplasmic spaces of root cells and their oxidative degradation within cells through use of a reactive oxygen staining technique. Collectively, these observations have suggested that plants are engaging in a process of microbivory in order to extract nutrients from microbes that colonize roots [45,46].

**Figure 1:**
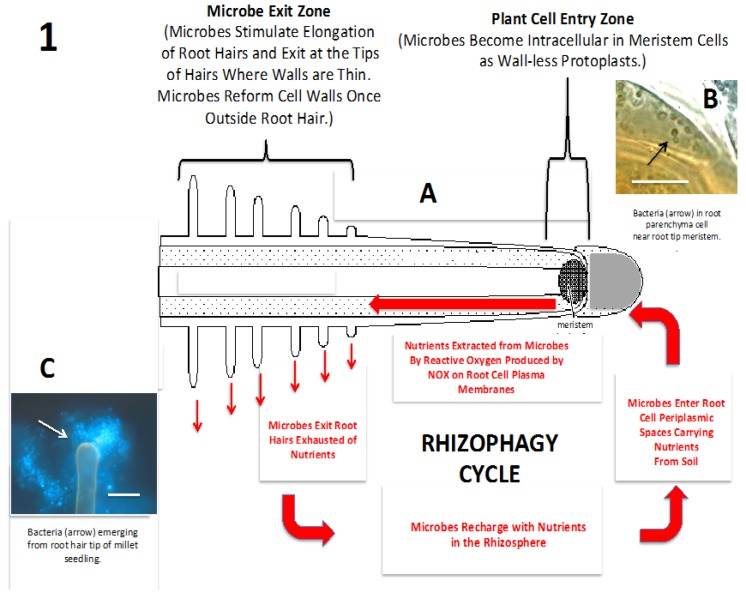
Diagrammatic representation of the rhizophagy cycle. (**A**) Diagram of the rhizophagy cycle showing microbes entering root cells at the root tip meristem and exiting root cells at the tips of elongating root hairs. Rhizophagy cycle microbes alternate between an intracellular endophytic phase and a free-living soils phase; soil nutrients are acquired in the free-living soil phase and extracted oxidatively in the intracellular endophytic phase; (**B**) Shows bacteria (arrow) in the periplasmic space of parenchyma cell near root tip meristem of an Agave sp. seedling (bar = 20 µm; stained with 3,3-diaminobenzidine followed by aniline blue); (**C**) Bacteria (arrow) emerging from root hair tip of grass seedling (bar = 20 µm; stained with fluorescent nucleic stain SYTO 9).

**Figure 2:**
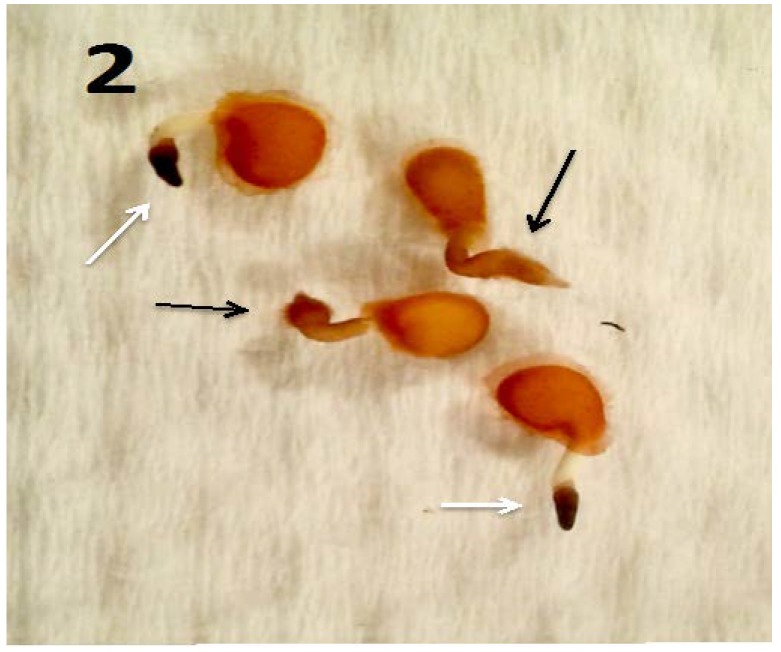
Tomato seedlings (3 days post germination) with *Micrococcus luteus* (black arrows) and without *M. luteus* (white arrows; stained with 3,3-diaminobenzidine). The endophyte-free seedlings failed to form root hairs and showed higher presence of reactive oxygen in root tips as is evidenced by deep brown color.

**Figure 3:**
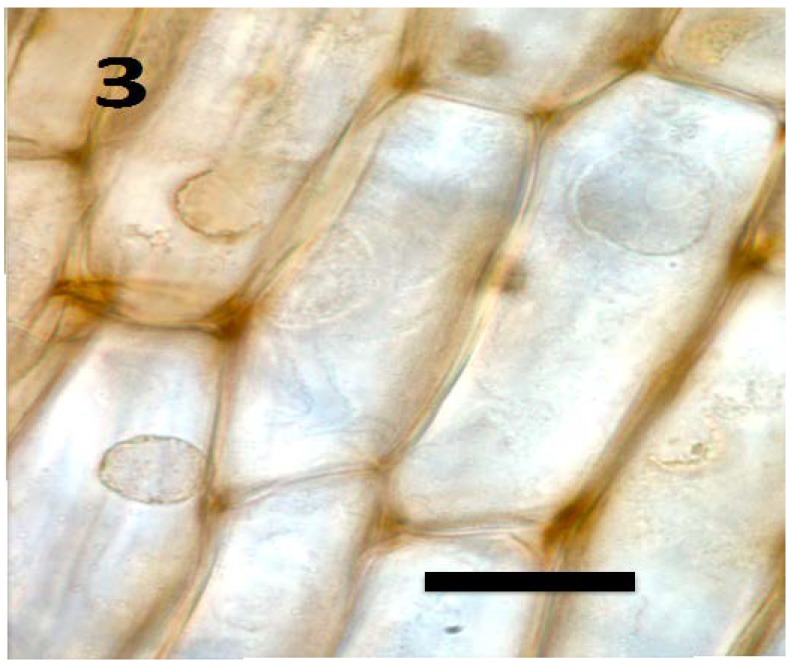
Root parenchyma cells of axenic tomato seedling showing absence of bacteria in cells (bar = 25 µm; stained with 3,3-diaminobenzidine followed by aniline blue).

**Figure 4:**
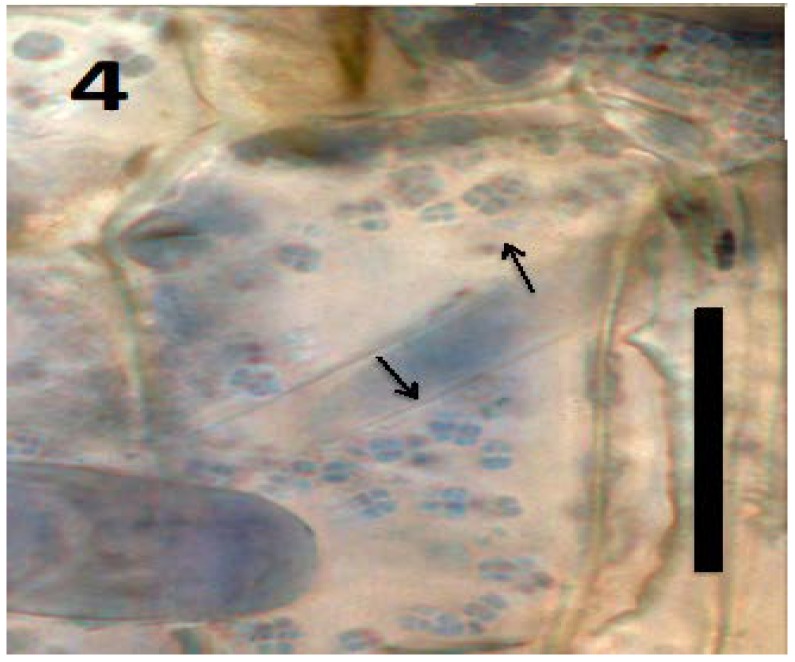
Parenchyma cell of tomato seedling inoculated with *Micrococcus luteus* showing tetrads of the bacterium (arrows) in periplasmic space of parenchyma cell (bar = 20 µm; stained with 3,3-diaminobenzidine followed by aniline blue).

**Figure 5:**
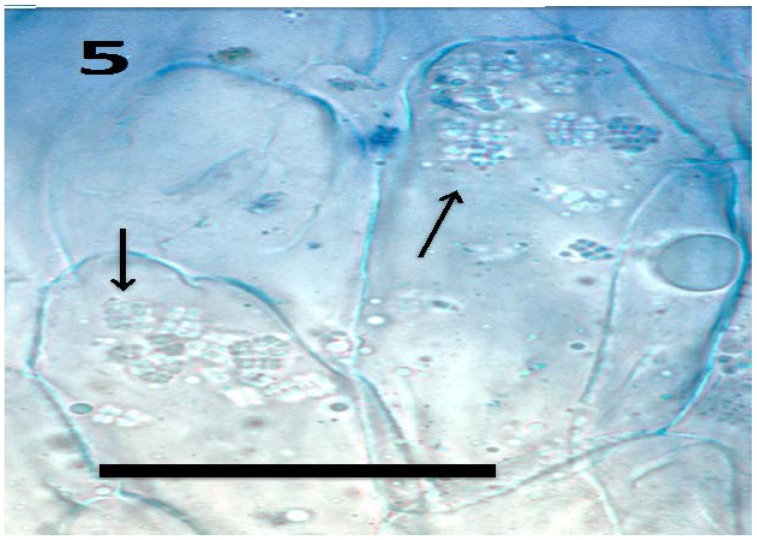
Tomato seedling root epidermis showing internal presence of tetrads (arrows) of *Micrococcus luteus* (bar = 20 µm; stained with aniline blue).

**Figure 6:**
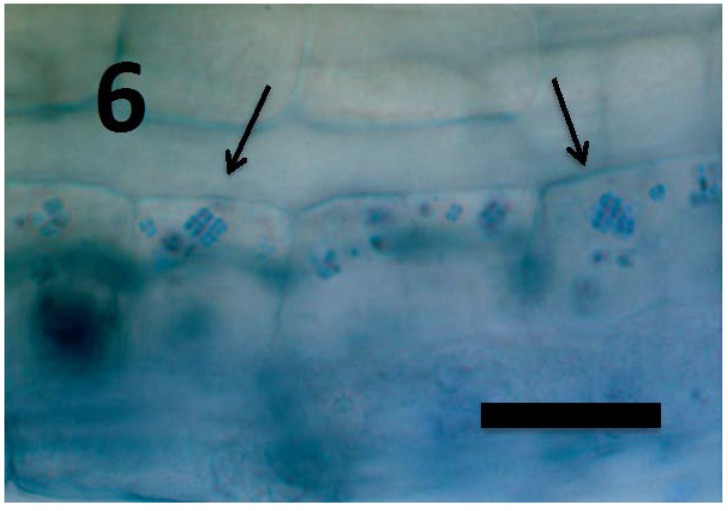
*Rumex crispus* seedling root parenchyma cells near root tip meristem showing internal presence of *Micrococcus luteus* tetrads (arrows; bar = 25 µm; stained with 3,3-diaminobenzidine followed by aniline blue).

**Figure 7:**
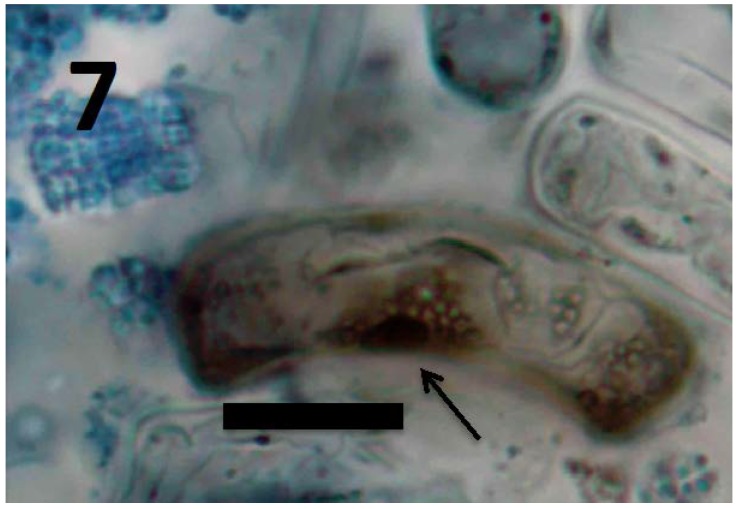
Root cells of *Daucus carota* seedling showing cluster of L-forms of *M. luteus* (arrow; bar = 20 µm; stained with 3,3-diaminobenzidine followed by aniline blue).

**Figure 8:**
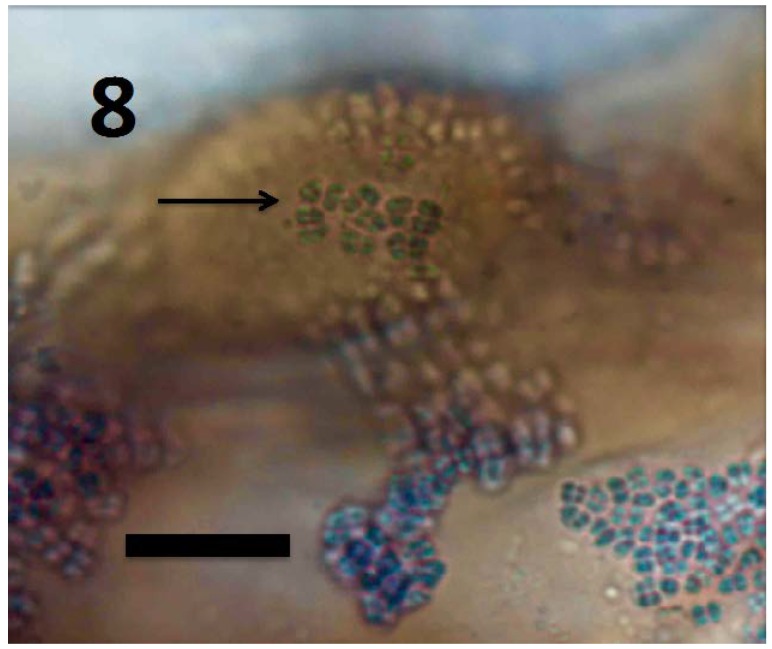
Root hair initial of *Rumex crispus* seedling showing *M. luteus* exiting root hair tip (arrow) and spilling off the sides of the root hair initial (bar = 10 µm; stained with 3,3-diaminobenzidine followed by aniline blue).

**Figure 9:**
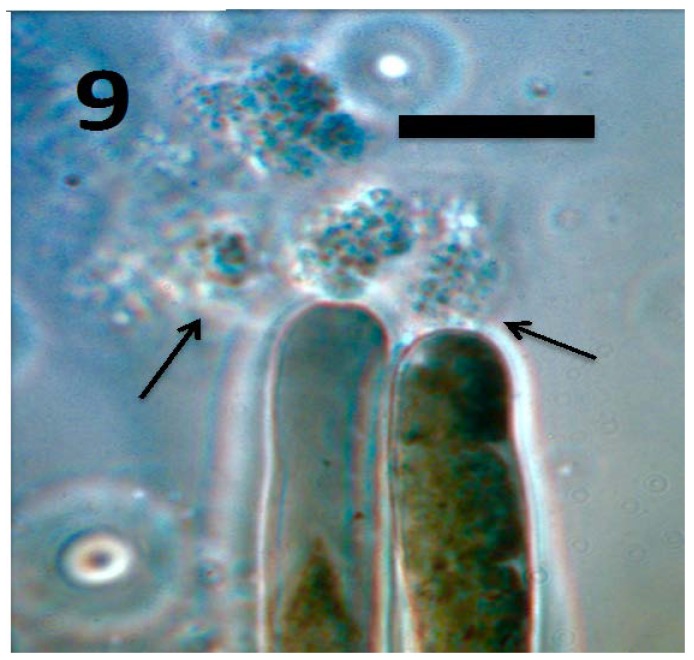
Root hairs of tomato seedling showing emergence of tetrads of *M. luteus* (arrows) from hair tips (bar = 20 µm; stained with 3,3-diaminobenzidine followed by aniline blue).

**Figure 10:**
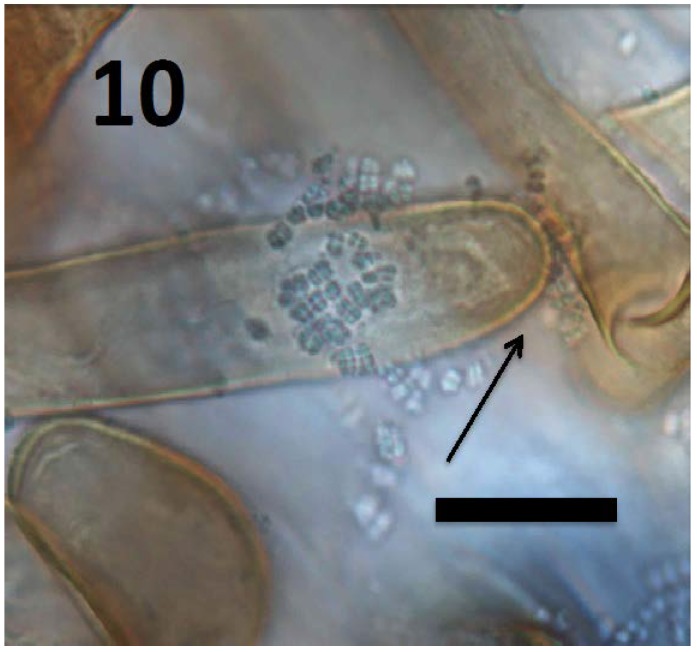
Root hair of *Rumex crispus* seedling showing tetrads of *M. luteus* (arrow) emerging from hair tip (bar = 20 µm; stained with 3,3-diaminobenzidine followed by aniline blue).

**Figure 11:**
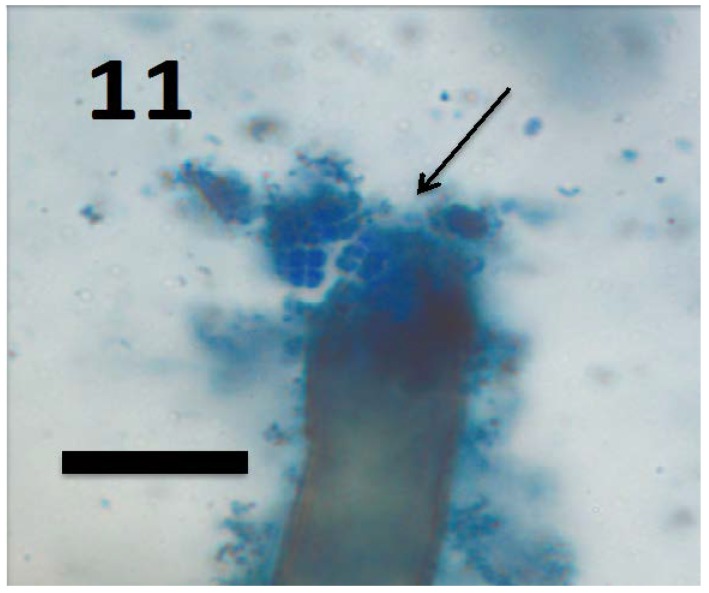
Root hair of seedling of grass *Cynodon dactylon* showing tetrads of *M. luteus* (arrow) emerging from ruptured root hair tip (bar = 15 µm; stained with 3,3-diaminobenzidine followed by aniline blue).

**Figure 12:**
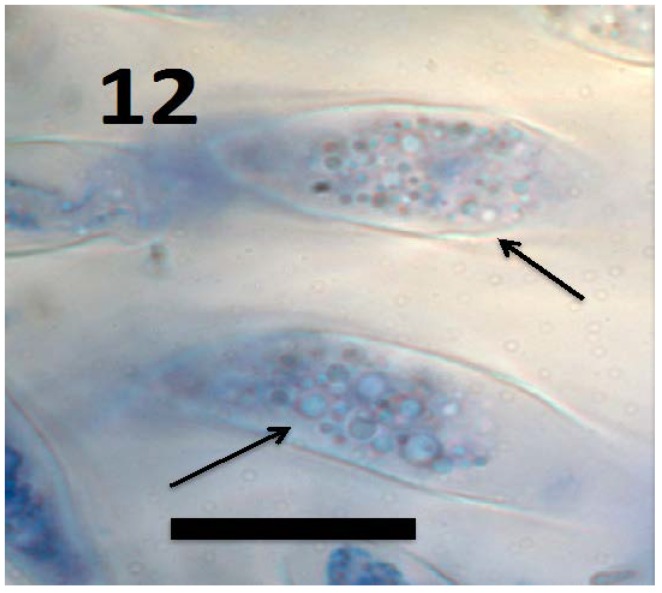
L-forms of *Bacillus amyloliquefaciens* (arrows) in root cap cells of grass *Urochloa ramosa* (bar = 15 µm; stained with 3,3-diaminobenzidine followed by aniline blue).

**Figure 13:**
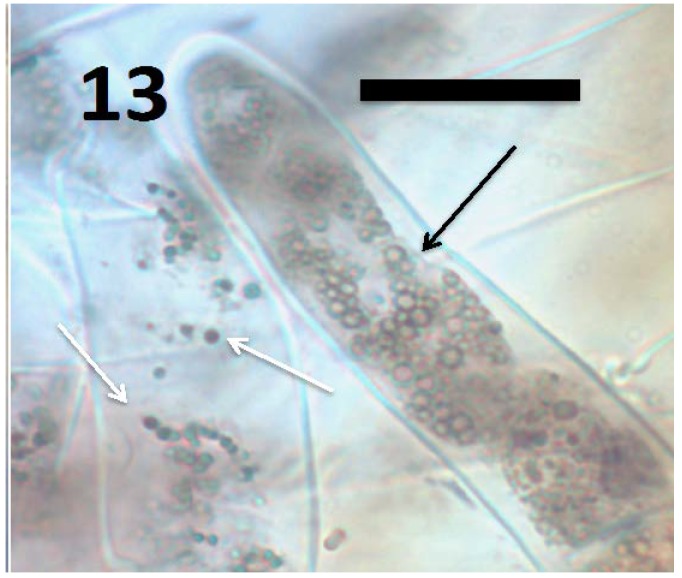
Root hairs of grass *Cynodon dactylon* seedling showing *B. amyloliquefaciens* (arrows) in hair (stained with 3,3-diaminobenzidine followed by aniline blue). The smaller blue-staining spherical structures (white arrows) are L-forms with cytoplasmic proteins intact; while larger spherical structures (black arrows) are oxidized L-forms that are swollen and lack cytoplasmic proteins, and as a consequence do not stain blue internally (bar = 20 µm).

**Figure 14:**
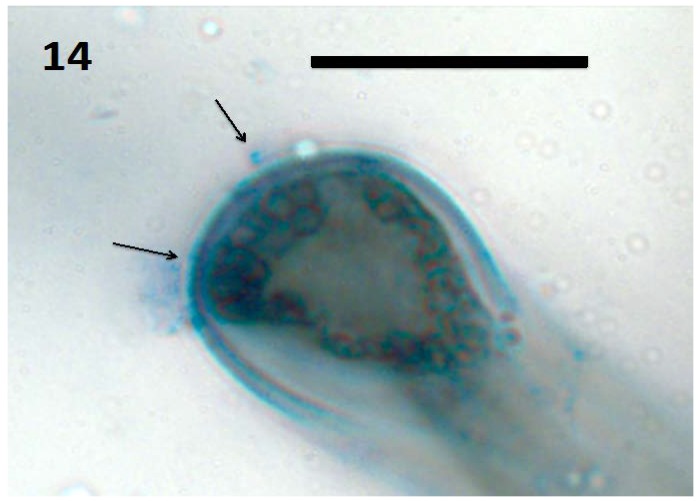
Tomato seedling root hair tip showing spherical L-forms of bacteria (arrows; bar = 20 µm; stained with 3,3-diaminobenzidine followed by aniline blue).

**Figure 15:**
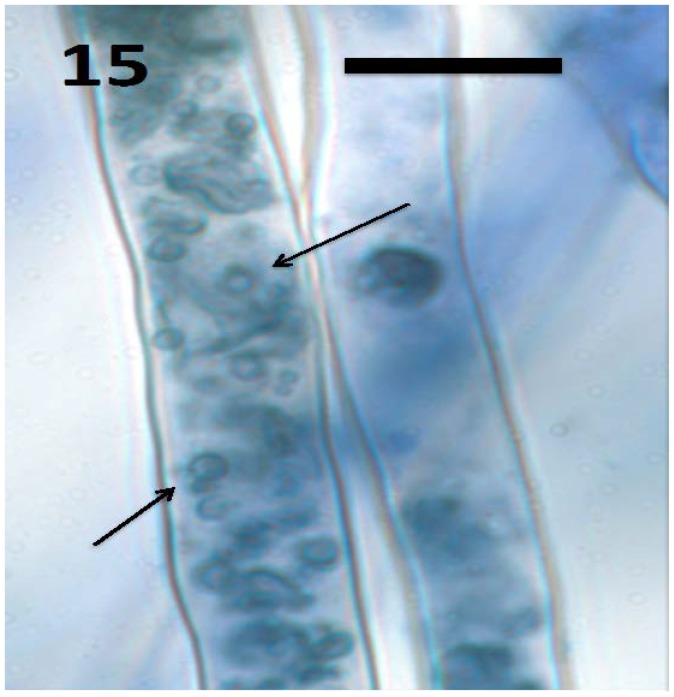
Root hairs of *Froelichia gracilis* seedling showing internal wall-less cells (mycosomes) of fungus *Aureobasidium pullulans* (arrows; bar = 15 µm; stained with 3,3-diaminobenzidine followed by aniline blue).

**Figure 16:**
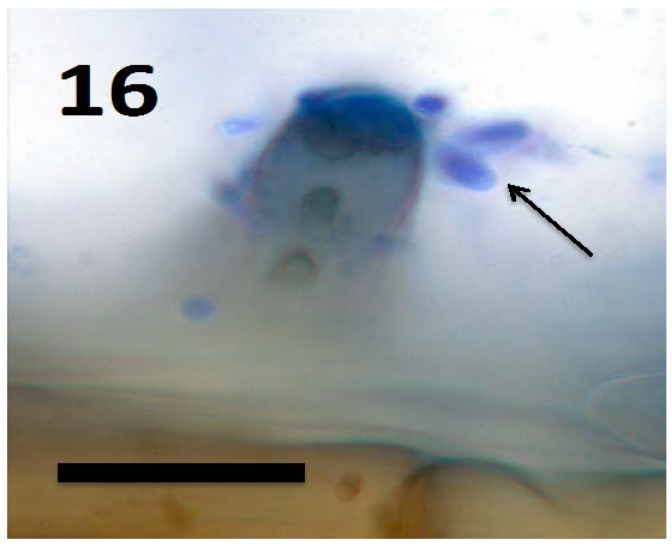
Root hair tip of *Amaranthus viridis* seedling showing yeast cells of *Aurobasidium pullulans* (arrows) exiting from the tip of the hair and brown staining walled yeast cells within the hair (bar = 15 µm; stained with 3,3-diaminobenzidine followed by aniline blue).

**Figure 17:**
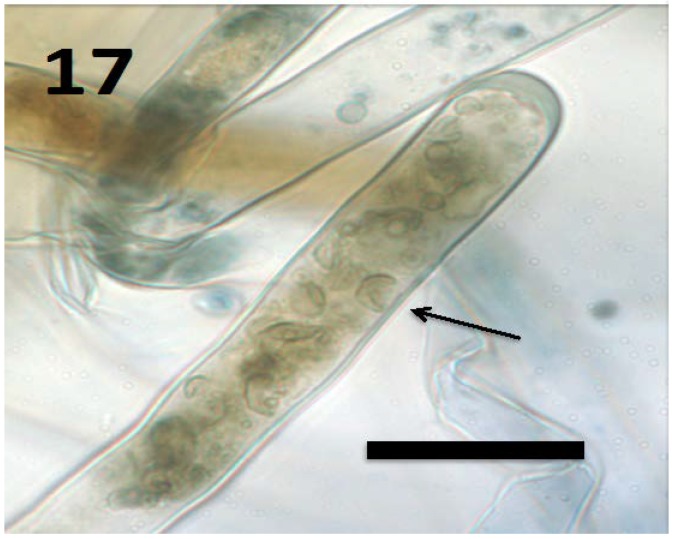
Root hair of *Froelichia gracilis* seedling showing internal yeast cells (collapsed mycosomes) of *Aureobasidium pullulans* (arrow; bar = 15 µm; stained with 3,3-diaminobenzidine followed by aniline blue).

**Figure 18:**
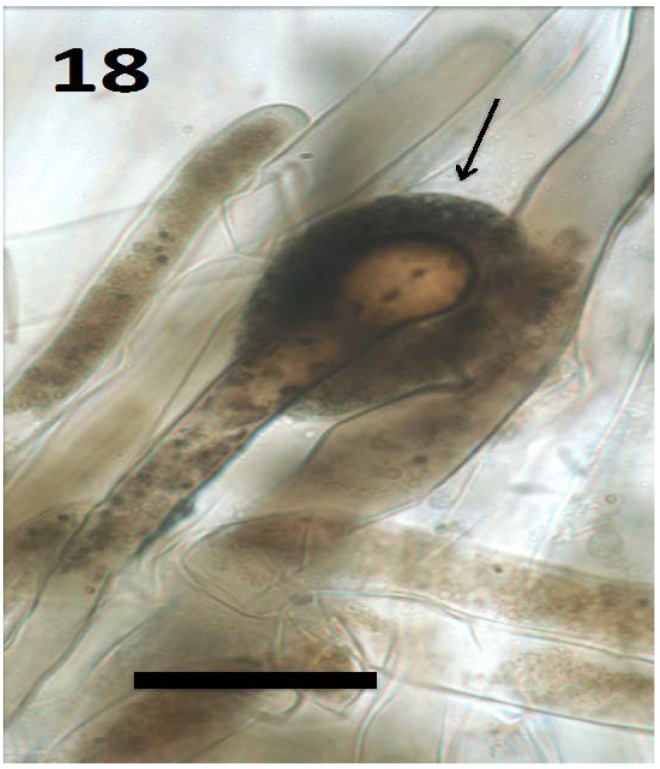
Root hair of seedling of *Froelichia gracilis* showing cap (arrow) composed of compacted yeast cells (bar = 30 µm; stained with 3,3-diaminobenzidine followed by aniline blue).

Over the past several years, we have conducted experiments using various host species and endophytic bacteria to elucidate how the plant microbivory process works in plant roots [46,47,48,49,50,51,52,53,54,55]. In this paper, we describe features of the microbivory process and hypotheses with regard to how the process works. We hypothesize that many plants, perhaps all plants, acquire some nutrients directly from symbiotic microbes by a process we term the ‘rhizophagy cycle’ (Figure 1). In the rhizophagy cycle symbiotic microbes (often seed transmitted bacteria) alternate between an intracellular/endophytic phase and a free-living soil phase. We hypothesize that microbes acquire soil nutrients in the free-living soil phase and that those nutrients are extracted from microbes oxidatively in the intracellular/endophytic phase. We also discuss proposed mechanisms plants employ to manipulate symbiotic microbes to transport nutrients from the soil into root cell periplasmic spaces, extract nutrients through oxidation and deposit surviving microbes exhausted of their nutrients back into the rhizosphere through the tips of elongating root hairs (Figure 2, Figure 3, Figure 4, Figure 5, Figure 6, Figure 7, Figure 8, Figure 9, Figure 10, Figure 11, Figure 12, Figure 13, Figure 14, Figure 15, Figure 16, Figure 17 and Figure 18).

## 2. The Symbiotic Bacteria

In our experiments, we employed microbes that were transmitted within or on the surfaces of seeds of several species of plants [46,47,48,56,57]. The bacteria that functioned in the rhizophagy cycle in in vitro experiments include species of genera of Gram-negative bacteria: Alpha-proteobacteria *Bosea*, *Methylobacterium*; Beta-proteobacteria *Achromobacter* and *Burkholderia*; Gamma-proteobacteria *Acinetobacter*, *Klebsiella, Pantoea*, *Pseudomonas* and *Micrococcus*; and Gram-positive Bacilli *Bacillus* and *Paenibacillus*; and Actinobacteria *Curtobacterium* and *Microbacterium*. Bacteria that function in the rhizophagy cycle belong to diverse classes of bacteria and it seems likely that any bacterium that colonizes roots and can be induced to enter root cells may be involved in the process [46,47,48,50,55].

## 3. Plant ‘Farming’ of Rhizosphere Microbes

Plants produce a root exudate zone adjacent to and just behind root tip meristems [58]. Plants secrete carbohydrates, amino acids, vitamins and organic acids into this zone [59]. The literature suggests that plants alter numbers and diversity of microbes on root surfaces and in the rhizosphere through secretion of exudates [60]. Plants are known to increase secretion of exudates in nutrient limiting soils, likely leading to increased microbial activity around roots and increased ‘microbial mining’ for nutrients [59]. Root exudates attract microbes that will grow in the root exudates [58,61]. In this sense root exudates act as signal molecules that attract a diverse community of microbes to the exudate zone and biofilm around the root tip meristem [61,62,63]. Through the continued secretion of root exudates plants are cultivating microbes and when nutrients are scarce plants increase cultivation of microbes by producing more exudates [59]. The response of plants to increase density and diversity of the microbial community around roots by secreting more root exudates in nutrient limiting situations is consistent with the hypothesis that the root associated microbes function in nutrient acquisition. Through the secretion of root exudates, plants may be considered to be ‘farming’ microbes.

## 4. Nutrients Transferred to the Host Plant

Some of the bacteria (e.g., *Burkholderia* and *Klebsiella*) involved in the rhizophagy cycle have been frequently found to fix atmospheric nitrogen, while others are sometimes or rarely reported as nitrogen fixing [64]. For bacteria involved in the rhizophagy cycle, it is unlikely that any nitrogen fixation occurs while they are in tissues of plant roots because bacteria are exposed to high levels of reactive oxygen in roots and nitrogenases are rapidly degraded by oxygen [43,44,64,65]. Any nitrogen fixation activities by rhizophagy cycle bacteria would likely occur in the free-living soil phase where bacteria grow in the rhizosphere in low oxygen conditions. Consistent with this, Roley et al. [31] found that a nitrogen-fixing *Burkholderia* only showed evidence of active nitrogen fixation in roots of switchgrass (*Panicum virgatum*) after root tissues senesced. On the other hand, it seems logical that bacteria could deliver nutrients to plants via the rhizophagy cycle without nitrogen fixation occurring; instead bacteria need only be capable of obtaining nutrients in the rhizosphere by scavenging of nutrients from soils, bacteria, fungi or plants. The capacity of some bacteria (including species of *Bacillus*) to produce and secrete resistant proteases may enable these bacteria to scavenge nitrogen from other microbes by degrading and absorbing their proteins in the rhizosphere [66]. Irizarry and White [57] showed that addition of *Bacillus amyloliquefaciens* to cotton seedlings growing in soil resulted in increased growth and an increased expression of nitrate transporter genes. In isotope tracking experiments using grass seedlings both with and without bacteria, grown in agarose amended with 15N-labeled proteins, White et al. [55] showed that presence of the bacteria on seedlings resulted in acquisition of approximately 30% more nitrogen by grass seedlings than in seedlings without bacteria. However, in that experiment, it was not possible to determine what proportion of the nitrogen was obtained from absorption of microbe-mineralized nitrogen in media around roots, versus from direct degradation of microbes within or on surfaces of roots. Hill, Marsden and Jones [67] conducted isotope-tracking experiments to assess the flow of nitrogen into wheat plants from direct consumption of microbes versus inorganic soluble nitrogen. These authors concluded that plant consumption of microbes was occurring but that the rate of movement of nitrogen through rhizophagy was one to two orders of magnitude slower than soluble inorganic nitrogen. Hill, Marsden and Jones [67] did not examine nutrient transfer to plants other than nitrogen. Thus, no work has been done to date to identify the particular nutrients that plants acquire from rhizophagy. It seems probable that plants would not be internalizing and degrading symbiotic microbes unless critical nutrients were being obtained from consumption of those microbes.

## 5. Rhizophagy Microbes as Carriers of Micronutrients

Through the activities of some symbiotic microbes, nutrients (e.g., phosphates, organic nitrogen) may be solubilized in the rhizosphere [68]. Solubilized nutrients may be absorbed by root hairs into roots. However, bacteria also efficiently scavenge nutrients in the rhizosphere (soil) and sequester difficult to obtain micronutrients (including boron, cobalt, copper, iron, manganese, magnesium and zinc) using biogenic ligands with a high affinity for metals called siderophores [69]. Root associated bacteria are often motile and capable of moving out away from the plant root in order to acquire soil nutrients—and of returning to the plant to acquire additional carbon and other root exudate nutrients. Bacteria would appear to be ideal carriers of micronutrients that are required for plant growth and development. Through the process of rhizophagy, all of the nutrients acquired or produced by bacteria could be oxidatively extracted from them. Bar-Ness et al. [70] showed that a pseudomonad was responsible for transporting iron from soil to dicot and monocot roots to support plant growth. Bar-Ness et al. [71] showed that in cotton (*Gossypium* sp.) iron was acquired directly from bacteria at the root tip meristems. In the process of rhizophagy, it is at the root tip meristems that bacteria enter plant cells and are exposed to reactive oxygen that likely extracts nutrients from the intracellular bacteria—inducing electrolyte leakage of bacteria and making nutrients available for absorption into plant root cells. Further work is needed to determine precisely which nutrients may be obtained by plants from the direct consumption/oxidation of microbes.

## 6. Balance between Microbe-Oxidation Susceptibility and Resistance

Experiments using bacterial endophytes *Pseudomonas* spp. (from seeds of grass *Phragmites australis*) and *Micrococcus luteus* (from seeds of *Lycopersicum esculentum*) that differed in resistance to reactive oxygen degradation due to their relative capacities to produce antioxidants (carotenoids, catalases, peroxidases and superoxide dismutase) have suggested that bacteria that participate in the rhizophagy cycle may be matched to their particular host plants. More specifically, rhizophagy bacteria must be degradable by levels of reactive oxygen produced by their hosts. *Pseudomonas* spp. that stimulated growth of several grasses [47,49] were observed entering root cells and then swelling and losing protein content as they were degraded. These same bacteria were not observed to degrade when they were put into the seedlings of dandelion (*Taraxacum officionale*) [47,49]. *Micrococcus luteus*, a tomato endophyte, was shown to stimulate growth of seedlings of tomato (Figure 2). However, inoculation of *Micrococcus luteus* into the seedlings of several other plant species, including grasses (*Festuca arundinaceae* and *Poa annua*), carrot (*Daucus carota*), curly dock (*Rumex crispus*) and Japanese knotweed (*Fallopia japonica*) resulted in internal colonization of root cells and suppression of root growth. Examination of roots of these species bearing *Micrococcus luteus* showed that bacteria colonized the interior of root cells, and elicited reactive oxygen production, but did not degrade within root cells, instead copiously replicating in seedling roots (Figure 4, Figure 5, Figure 6, Figure 7, Figure 8, Figure 9, Figure 10 and Figure 11) to exit seedlings and accumulate *en masse* around roots. This could be explained by the increased capability of *Micrococcus luteus* to withstand reactive oxygen levels in hosts other than tomato. Mohana, Thippeswamy and Abishek [72] showed that carotenoids produced by *Micrococcus luteus* protect the bacterium from oxidation. Ohwada et al. [73] showed that *Micrococcus luteus* has 91 times more catalase activity and 20 times more peroxidase activity than *Pseudomonas*. The results of our experiments with pseudomonads and *Micrococcus* seem to indicate that microbes that establish a symbiosis with plants in the rhizophagy cycle must be susceptible to degradation by the host in the intracellular phase. In this study, the pseudomonads were compatible with their grass hosts and were degraded (or some of them were degraded) within roots. The degradable pseudomonads may thus provide a source of nutrients to their grass host plants, resulting in growth stimulation. *Micrococcus* was resistant to degradation by several hosts and likely did not provide nutrients. Further, because *Micrococcus luteus* was resistant to degradation in the root several hosts, the numbers of bacteria in cells could not be regulated and likely overwhelmed the seedlings due to bacterial overload. This emphasizes that plants and their symbiotic microbes may be adapted optimally to maximize benefits of the symbiosis. In an optimal rhizophagy cycle relationship, microbes would enter plant roots and some would be degraded oxidatively, keeping intracellular microbe numbers in check, while survivors would exit roots to rejoin soil populations.

## 7. Mechanisms for Microbe Entry into Roots

### 7.1. Endocytosis Hypothesis to Explain Microbe Entry into Root Meristem Cells

Paungfoo-Lonhienne et al. [39] proposed that movement of microbes into plant cells is a form of endocytosis where plant derived cell-wall degrading enzymes play a role in loosening cell walls to permit entry of microbes into plant root cells. Experiments using *Arabidopsis thaliana* and *Lycopersicon esculentum* inoculated with *Escherichia coli* showed that plants expressed higher levels of cellulases than un-inoculated controls. These authors also found that bacteria on surfaces of root cells were sometimes covered by a matrix composed of cellulose fibers as evidenced by immuno-histochemical experiments. It was proposed that coating microbes in a matrix could be an early stage of endocytosis into root cells. Further, Paungfoo-Lonhienne et al. [39] reasoned that movements in the root cell cytoskeleton were consistent with the hypothesized active endocytosis of microbes by root cells. An active endocytosis mechanism for internalizing microbes into root cells is logical and cannot be ruled out; however, another mechanism involving manipulation of microbe virulence is possible based on experiments that we have conducted.

### 7.2. Hypothesized Role of Short-Chain Fatty Acids in Induction of Bacteria to Enter into Root Meristem Cells

Bacteria growing in biofilms on the root surface and in the rhizosphere anaerobically ferment carbohydrates present in root exudates to form short-chain fatty acids (SCFAs). Butyric and propionic acids are among the SCFAs fermented by bacteria associated with roots [74]. In other research [75,76], butyric and propionic acids in animal intestines have been shown to act as signal molecules or inhibitors of some bacteria and yeasts. When SCFAs are in high concentration in biofilms, bacteria and yeasts (e.g., *Salmonella* spp. and *Candida* spp.) remain in the biofilm phase but when butyric and propionic acid concentrations fall, virulence genes in microbes are up-regulated and microbes parasitize epithelial tissues in the walls of the animal’s gut [75,76]. Butyric acid and its derivatives are now widely being used to suppress gut parasitism by bacteria in agricultural/industrial animal production [77,78]. In isotope tracking experiments, Tramontano and Scanlon [79] showed that root meristem cells absorbed butyric and propionic acids and transported these compounds to the cell nucleus where the root cell responded by slowing cell division. Lanzagorta, de la Torre and Aller [80] found the 2 mM concentration of butyrate reversibly slowed the cell cycle of *Allium cepa* root tip meristem cells. Why plant cell division in the meristem slows is unknown, but it shows that the plant may respond to presence of the microbes around the meristem. The active absorption of these SCFAs by root meristem cells, may stimulate bacteria in the biofilm around the root meristem to infect the thin-walled meristem cells in a comparable way microbes in intestines infect intestinal epithelial tissues. Thus, absorption of SCFAs by the root meristem is a possible ‘rhizophagy trigger,’ essentially inducing microbes to parasitize the meristem cells. In this sense, the removal of organic acids from the microbial biofilm around root meristems may represent part of the symbiotic ‘cross talk’ between plant and microbe, comparable to interactions that occur in establishment of rhizobial symbioses [5].

### 7.3. Propionic Acid and Butyric Acid Experiment to Test SCFA Removal-Induced Infection of Meristem Cells

To test whether absorption of propionic and butyric acids from the bacterial biofilm by the root tip meristem is the trigger to initiate entry of bacteria into root meristem cells, we conducted experiments in which we grew seedlings of the grass *Poa annua* (inoculated with *Pseudomonas fluorescens*) on agarose (Sigma, Burlington, MA, USA) that contained 0, 2, 4, 6, 8, or 10 mM of butyric or propionic acids (Sigma, Burlington, MA, USA). After approximately 10 days, seedlings were stained for 15 h in a solution of 3,3-diaminobenzidine (DAB) (Sigma, Burlington, MA, USA) and counterstained using aniline blue (0.01%, aqueous; Sigma, Burlington, MA, USA), then examined microscopically for evidence of intracellular bacteria using a compound light microscope (Zeiss, Axioskop; Carl Zeiss Microscopy, Thornwood, NY, USA) [44]. In these experiments, we found that concentrations of SCFAs from 0–4 mM gradually reduced entry of bacteria into root cells and at 4 mM no bacteria could be seen within root cells [46,51,53]. These experimental results are consistent with the hypothesis that absorption of SCFAs from the bacterial biofilm around the root tip meristem is a trigger for intracellular invasion of bacteria into meristem cells. In our experiments, at 4 mM/L of SCFAs in the agarose, the root meristem cells could not remove the SCFAs from the bacterial biofilm due to its constant replacement by additional SCFAs from the agarose. However, whether a mechanism of root cell endocytosis, as suggested by Paungfoo-Lonhienne et al. [39], or active infection by microbes triggered by removal of bacterial fermentation products by the root meristem, a combination of the two mechanisms, or another mechanism entirely, accounts for internalization of microbes into root cells will require additional investigation.

## 8. Loss of Cell Walls by Bacteria on Entry into the Periplasmic Space of Root Cells

Microscopic examination (Figure 4, Figure 5, Figure 6, Figure 7, Figure 8, Figure 9, Figure 10 and Figure 11) of a diverse selection of seedlings of grasses (*Cynodon dactylon* and *Poa annua*) and dicots (*Rumex crispus*, *Daucus carota*, *Fallopia japonica* and other species) infected by *Pseudomonas* spp. or *Micrococcus luteus* shows that bacteria initially enter root meristem cells as walled cells—pseudomonads are rod-shaped and *Micrococcus luteus* cells are spherical and in tetrads. In the periplasmic space of root cells, bacteria lose cell walls and form spherical L-forms (Figure 12, Figure 13 and Figure 14) [81]. Likely, it is exposure of bacteria to reactive oxygen, constitutively produced in the meristematic cells, that triggers bacteria to become wall-less L-form bacteria [82]. The reactive oxygen may damage bacterial membrane wall synthesis enzymes that results in formation of the wall-deficient bacteria. L-forms can also be induced experimentally in bacteria by exposure to antibiotics that inhibit cell wall synthesis by bacteria [81], or spontaneously in plant and animal tissues [81]. L-form bacteria have been shown to occur in both animal and plant tissues and have been associated with symbiosis and disease [83,84,85]. In the L-form phase bacteria do not divide regularly or possess defined cell sizes. Instead, bacteria often form chains of spherical cells that ‘bleb’ or ‘bud’ to smaller and smaller sizes.

## 9. Intracellular Bacteria Exit Roots to Re-Enter Soil Populations

Our previous observations suggest that bacteria exit root hairs at the elongating root hair tips (Figure 8, Figure 9, Figure 10 and Figure 11). When all bacteria have exited the hair through the tip, hair elongation stops. This suggests that root hair growth, at least in some cases, is a function of intracellular bacteria. It also suggests that root hairs may function as a mechanism to deposit bacteria exhausted of nutrients back out into the rhizosphere where they can acquire additional nutrients before returning to the root exudate zone. Confirmation of movement back into the soil is significant because it completes the cyclic process of ‘rhizophagy’—with the potential that intracellular bacteria may re-enter soil and eventually return to roots with nutrients that could support plant growth. A ‘rhizophagy cycle’ where roots continuously extract small amounts of minerals, vitamins, or other growth factors from endosymbiotic bacteria could be an important source of nutrients for plants that has been largely overlooked by plant scientists. The occurrence of a rhizophagy process that is widespread in plants could explain how some bacteria that are not able to fix nitrogen themselves nevertheless may show significant enhancements in plant growth promotion [32].

## 10. Do Fungi Also Function in the Rhizophagy Cycle?

In a recent study, we isolated the fungus *Aureobasidium pullulans* (Ascomycota) from seeds and seedlings of the plant *Froelichia gracilis* (Amaranthaceae). We also isolated the endophytic yeast *Rhodotorula* sp. from *Abrus precatora* (Fabaceae). Experiments using *A. pullulans* and *Rhodotorula* sp. demonstrated that these yeasts internally colonized seedling roots of several plant species, entering into root cells and locating in the periplasmic spaces of root parenchyma [46]. Initial colonization appears to occur at the root tip meristem based on presence of fungal cells in periplasmic spaces of parenchyma cells in outer cell layers of the root behind the tip meristem and in root hairs at all stages of development. The fungi also have the capability to form wall-less protoplasts called ‘mycosomes’ that can bud sequentially to form chains within plant cells [86]. In our experiments, we observed both walled fungal cells (Figure 18) and apparent wall-less mycosomes (Figure 15 and Figure 17) within seedling root cells. The fungi were also seen to exit root cells at the tips of elongating root hairs (Figure 16), accumulating around hair tips as masses of yeast cells, often forming caps on tip of the hairs (Figure 18). Our experiments using both yeasts show that fungi follow the same path through plant roots as seen in bacteria involved in the rhizophagy cycle. These yeasts appear to cycle between an intracellular endophytic phase and a free-living soil phase. It seems reasonable to hypothesize that nutrients could be extracted from fungi oxidatively when they are in the protoplast mycosome phase. Atsatt and Whiteside [86] observed mycosome formation in several additional species of fungi in phyla Ascomycota, Basidiomycota and Zygomycota, suggesting that other fungi may also possess the capability to become internalized in plant root cells. Much more work is needed to evaluate the potential benefits of intracellular fungi to their host plants.

## 11. Non-Nutritive Functions of Rhizophagy Microbes

### 11.1. Modulation of Plant Development

Experiments using seedlings of grasses and other plant species have demonstrated that some, or perhaps all, microbes that internalize in plant cells modulate development of the seedlings. Seedlings from rigorously surface-disinfected seeds using sodium hypochlorite to remove all surface microbes invariably are diminished developmentally, often showing loss of root gravitropic response (roots fail to grow downward) and reduced or no root hair formation. Re-inoculation of the symbiotic bacteria onto axenic seeds restores normal root growth in seedlings [43,48,49,50]. Thus, it is evident that plants appear to rely on symbiotic microbes to modulate development. Modulation of plant development may be considered to be a basic function of microbes in the rhizophagy cycle. The hypothesized mechanisms for modulation of plant development may relate to microbial production, or removal of, plant hormones [36,38,53,87,88].

### 11.2. Enhancement in Oxidative Stress Tolerance in Host Plants

Entry of microbes into root cells is generally accompanied by increased production of defensive reactive oxygen by the root cells [47]. Increased production of reactive oxygen may cause the host to increase production of antioxidants (e.g., superoxide dismutase, peroxidases, catalases) and oxidative stress-related genes to reduce the negative effects of reactive oxygen on the host itself. The direct association between the amount of reactive oxygen secreted by the host and its resistance to oxidative stress has not been verified but endophytic microbe colonization of plants frequently provides hosts with increased tolerance to abiotic stresses (e.g., drought, heavy metals, salinity and high temperatures) and biotic stresses (e.g., diseases, herbivory) and the underlying mechanism in many cases has been resistance to oxidative stress [20,21,22,56,57,89,90,91,92].

### 11.3. Enhanced Disease Resistance Due to Endophytic Microbes

Some of the microbes that become intracellular in plant roots have been shown to inhibit pathogenic fungi in the soil or in the plant [25,26,49,50,93,94]. Disease protection may be the result of induced systemic resistance (ISR) where the endophyte causes the plant to up-regulate its disease resistance genes making plants more resistant to pathogens [26,95]. However, some of these microbes also have direct inhibitory activity on pathogenic fungi. Soares et al. [27] showed that a *Bacillus* endophyte of English ivy (*Hedera helix*) produced antifungal lipopeptides that directly inhibited growth of the pathogenic fungus *Alternaria tenuisima* and protected the host from disease. Pseudomonad endophytes of plants also venture into the soil and colonize potential fungal pathogens, repressing their growth and reducing virulence [47]. Pseudomonads are known to produce antifungal compounds including 2,4-diacetylphloroglucinol, pyoluteorin, pyrrolnitrin and hydrogen cyanide; these and/or other compounds may inhibit growth of potential pathogens and/or alter their behavior in reducing growth and virulence [96].

### 11.4. Endophyte-Mediated Suppression of Competitor Plant Species

Microbes involved in the rhizophagy cycle intimately associate with plant root tissues and cells, growing around the root tip meristems and passing through cell walls where they come into direct contact with root cell plasma membranes. We hypothesize that this intimate association requires that the microbe must be subject to control by the plant cells. If the intracellular microbes are resistant to degradation by host-produced reactive oxygen due to production of antioxidants the microbe may replicate itself excessively at the expense of the host cell and host growth may be reduced. If microbes produce metabolites such as hydrogen cyanide or other compounds that restrict respiration or other functions of the host cell, plant growth may again be compromised. Thus, it seems reasonable that hosts and rhizophagy cycle endophytes are adapted to maximize benefits to both hosts and microbes. Colonization of rhizophagy cycle endophytes into hosts to which they are not adapted may result in ‘endobiome interference,’ where endophytes reduce growth and competitiveness of hosts and perhaps interfere with the growth promotional activities of native endophytes [53]. In some of our experiments ‘endobiome interference’ may have occurred. For example, experiments we conducted using seed-transmitted pseudomonads from the grass *Phragmites australis* showed that the bacteria stimulated growth of several grasses but suppressed growth and increased mortality in seedlings of dandelion (*Taraxacum officionale*) and curly dock (*Rumex crispus*) [47]. In other experiments [53], we showed that endophytic yeasts *Rhodotorula* sp. and *Aureobasidium pullulans* and bacteria *Micrococcus luteus* and *Paenibacillus* sp. from seedlings of plant species *Abrus precatorius*, *Froelichia gracilis*, *Lycopersicum esculentum* and *Poa annua*, respectively, suppressed root growth and increased mortality of seedlings of dandelion (*Taraxacum officionale*), curly dock (*Rumex crispus*) and clover (*Trifolium repens*). Here, microbes that were most inhibitory to seedling growth were those that were resistant to reactive oxygen due to production of antioxidants—and likely internal replication of microbes could not be controlled by the inhibited seedlings, resulting in replication of endophytes at the expense of host growth. We do not know the mechanisms by which endophytic colonization of non-hosts led to increased seedling mortality. Endobiome interference could be a factor that affects competition between plant species in natural plant communities—with endophytes serving as a means to suppress the growth of competitor plant species [47]. Additional experiments are needed to determine whether ‘endobiome interference’ occurs in natural plant communities.

## 12. Conclusions

The ‘rhizophagy cycle’ is a process where microbes alternate between an endophytic phase and a free-living soil phase. Rhizophagy microbes become intracellular in root cells by infecting cells at the root tip meristem. Microbes exist primarily as wall-less protoplasts (‘L-forms’ for bacteria and ‘mycosomes’ for fungi) in close association with host cell plasma membranes in roots. Root cell plasma membranes secrete reactive oxygen onto microbes and this may reduce replication of intracellular microbes and cause leakage of nutrients from microbes. Intracellular microbes trigger formation of root hairs on roots and they exit cells at the elongating root tips, reforming cell walls as they exit from root hairs. Plants appear to manipulate microbes in the rhizophagy cycle by: (1) stimulating bacterial growth around root tip meristems of seedlings by secretion of root exudates around the root tip; (2) triggering bacteria to enter into periplasmic spaces in root cells at the root-tip meristem by absorbing bacterial fermentation products including butyric acid, causing bacteria to up-regulate virulence/endoparasitism genes; (3) subjecting bacteria in periplasmic spaces to superoxide formed on root cell plasma membranes to extract nutrients from bacteria, and (4) depositing surviving intracellular bacteria back into the rhizosphere from the tips of elongating root hairs to maximize new nutrient acquisition by bacteria. Microbes engage in the rhizophagy symbiosis likely because they also benefit from the nutrients provided by host plants in terms of root exudates and nutrients that leak from root cells during the endophytic/intracellular phase. The rhizophagy symbiosis may be viewed as a mutualism involving an exchange of nutrients between the plant and microbe participants. Much of our research to date on the rhizophagy cycle has been done on seedlings, however future research is needed to confirm that the rhizophagy cycle also occurs at root tips of more mature plants. While ‘rhizophagy’ has been shown to increase movement of nitrogen into plants, we hypothesize that the real benefit of the rhizophagy cycle may be in acquisition of iron and other micronutrients from symbiotic microbes that sequester these soil nutrients using siderophores, although this must be proven. The biological implications of the rhizophagy cycle and microbes that are involved in it in terms of plant growth promotion and plant-plant interactions via symbiotic microbes are topics that require much additional research.
